# Susceptibility of Novel Promising Citrus Rootstocks to White Root Rot

**DOI:** 10.3390/plants11233388

**Published:** 2022-12-05

**Authors:** Juan M. Arjona-López, Frederick G. Gmitter, Estefanía Romero-Rodríguez, Jude W. Grosser, Aurea Hervalejo, Carlos J. López-Herrera, Francisco J. Arenas-Arenas

**Affiliations:** 1 Department of Agri-Food Engineering and Technology, Andalusian Institute of Agricultural and Fisheries Research and Training (IFAPA), “Las Torres” Center, Ctra. Sevilla-Cazalla de la Sierra km. 12.2, 41200 Seville, Spain; 2 Citrus Research and Education Center, Department of Horticultural Sciences, IFAS, University of Florida, Lake Alfred, FL 33850, USA; 3 Institute for Sustainable Agriculture, Spanish Research Council, Alameda del Obispo s/n, 14004 Cordoba, Spain

**Keywords:** citriculture, citrus diseases, crop protection, plant material, *Rosellinia necatrix*

## Abstract

Citrus is one of the most important fruit crops in Mediterranean countries such as Spain, which is one of the main citrus-producing countries worldwide. Soil-borne pathogens, such as *Rosellinia necatrix*, are relevant limiting biotic factors in fruit trees, due to their tricky management. This fungus is a polyphagous plant pathogen with worldwide distribution, causing white root rot in woody crops, including citrus trees in Spain. The objective of this study was to evaluate the tolerance of new plant material against *R. necatrix* infection. Therefore, plants of 12 different citrus rootstocks were inoculated with one *R. necatrix* isolate. During the assay, and periodically, above-ground symptoms and chlorophyll content were evaluated. At the end of the experiment, leaf area and plant biomass measures were obtained. Rootstocks B11R5T64 and B11R5T60 achieved the lowest disease incidence of symptoms and reduction of biomass, and were similar to their respective controls in chlorophyll content and leaf area. Carrizo citrange, CL-5146 and UFR-5 were the most affected rootstocks in symptoms and biomass reduction. This work provides information about *R. necatrix*-tolerant citrus rootstocks, which can constitute a new integrated, sustainable and effective long-term strategy to avoid white root rot.

## 1. Introduction

The citrus industry, composed of several fruit types, is one of the most economically important fruit production industries in Mediterranean and subtropical environments. In 2020, Spain ranked sixth in total production and is the top exporter of fresh citrus fruits in the world, with a total of 6.6 and 3.7 million tons, respectively [1]. 

*Rosellinia necatrix* Prill. (anamorph: *Dematophora necatrix* Hartig) is a soil-borne ascomycete with worldwide distribution, mostly in temperate and subtropical regions [2]. This fungus is a polyphagous plant pathogen with a host range comprising more than 340 plant species in 160 genera [3]. It causes white root rot disease with relevant economic losses in many crops and ornamental plants, especially in fruit trees [2,4,5,6]. In Spain, *R. necatrix* infects and affects important woody crops, such as apple, avocado, cherry, citrus, grapevine, loquat, mango, olive, peach, pear and/or plum. Additionally, this pathogen has been found in different Spanish agricultural-producing areas, including Valencia and Andalusia, which encompass the major citrus production regions in Spain [7,8,9,10,11].

The management of *R. necatrix* is difficult because this fungus can tolerate dry conditions and acidic soils, has a wide host range and extensive distribution in the soil, and is tolerant to many common fungicides [2]. Different strategies have been reported for controlling this disease in other crops, such as the following: cultural methods [12,13]; soil solarization [14,15,16]; antagonistic fungi, including *Trichoderma* spp. [16,17,18], *Entoleuca* sp. [19,20], non-pathogenic *R. necatrix* [21], *Crinipellis tabtim* or *Fusarium equiseti* [22]; antagonistic rhizobacteria, such as *Pseudomonas* spp. or *Bacillus* spp. [23,24,25]; mycoviruses (Reovirus, Megabirnavirus and Hypovirus) [26,27,28]; and integrated management with the use of antagonistic fungi plus antagonistic bacteria [29], antagonistic rhizobacteria, *Trichoderma* spp. or non-pathogenic *R. necatrix* plus fungicide [30,31,32]. Regarding chemical control, several chemical substances have been described in other woody crops (apple, avocado, grapevine or pear), which include thiophanate methyl, benomyl, formaldehyde, carbendazim, metham sodium and/or fluazinam [33,34,35,36,37]. However, environmental concerns, the presence of pesticide residues in food and the emergence of pathogen resistance [38,39,40] are reducing the use of synthetic compounds in European agriculture (Directive 2009/128/CE) [41]. Thus, these active substances are banned by European Union authorities [42], except metham sodium and fluazinam, which are not allowed to be used in Spanish citrus orchards [43]. In addition, the “farm to fork” strategy intends to reduce, by 50%, the use of chemical pesticides and to reach a situation where 25% of total farmland is under organic farming by 2030 [44].

On the other hand, the use of healthy tolerant rootstocks is widely reported as a long-term, effective and sustainable method to protect citrus orchards against other soil-borne pathogens, such as *Phytophthora* spp. [45]. Nevertheless, few research groups have directly evaluated the genetic resistance of rootstocks against *R. necatrix* in fruit trees. Previous research identified some tolerant candidates to this pathogen in apple, avocado, grapevine and persimmon [46,47,48,49,50,51,52]. In the case of citrus rootstocks, Sztejnberg and Madar [5] studied the influence on the host range for *R. necatrix* with four different rootstocks (Troyer citrange, *Poncirus trifoliata*, *Citrus macrophylla* and *C. aurantium*), in which, P. trifoliata displayed the highest tolerance response. Carrizo citrange (*C. sinensis* ‘Washington’ × *P. trifoliata*) is the most cultivated citrus rootstock in Spain, with a frequency of 61% [53] and there is no information about the response of Carrizo citrange to *R. necatrix*.

In addition, the Mediterranean basin is at risk of emerging diseases, such as Huanglongbing or citrus greening disease (HLB), which is described as the most devastating citrus disease worldwide [54], caused by three phytopathogenic bacteria species from the genus “*Candidatus* Liberibacter” [55,56]. Mainly, and naturally, HLB causal agents are transmitted by insect vectors, such as *Trioza erytreae* and *Diaphorina citri* [57,58,59,60]. Currently, *T. erytreae* is scattered among mainland citrus trees in Spain and Portugal, and has been since 2014 [61,62,63,64], while *D. citri* was detected in Israeli mandarin and orange orchards in August, 2021 [65].

Nowadays, citrus breeding programs include the development of new plant material with tolerance against HLB. To our knowledge, this new plant material has never been evaluated against endemic diseases in Spain, such as white root rot. The main aim of this study was, therefore, to characterize the response of new citrus rootstocks, originating from different breeding programs, to white root rot disease, following artificial inoculations with by *R. necatrix*.

## 2. Results

### 2.1. Identification of Fungal Isolate

The isolate (Rn452) used in this study was corroborated as *R. necatrix* by molecular methods, which showed a 100% identity with *R. necatrix*, using Blast analysis. The sequences were deposited in the GenBank database with the accession number OP482261.

### 2.2. Plant Symptoms and Responses of Different Citrus Rootstocks

**Figure 1:**
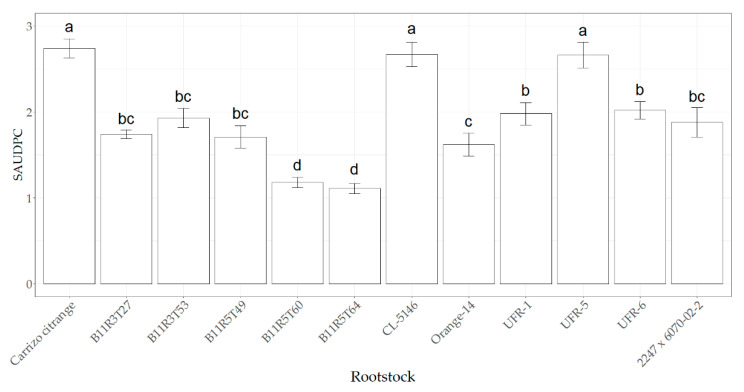
Mean standardized area under the disease progress curve (SAUDPC) ± standard error (SE) due to the effect of *R. necatrix* inoculation on aerial symptoms in 12 citrus rootstocks (Carrizo citrange, B11R3T27, B11R3T53, B11R5T49, B11R5T60, B11R5T64, CL-5146, Orange-14, UFR-1, UFR-5, UFR-6 and 2247 × 6070-02-2). Values in columns with different letters denote statistical differences among citrus rootstocks and between treatments assayed by LSD–Fisher’s test (*p* < 0.05).

The plant symptoms under the SAUDPC response displayed statistical differences among the citrus rootstocks and between the *R. necatrix* inoculation treatments (F_11,96_ = 20.89, and *p* < 0.001). First, non-inoculated citrus plants from all rootstocks did not show disease symptoms in the control treatment, with the symptom rate of healthy plants in all of them. In the inoculated treatment, Carrizo citrange, CL-5146 and UFR-5 showed the highest disease incidence with similar responses among them, which were significantly different, compared with the remaining rootstocks. On the other hand, B11R5T64 and B11R5T60 displayed the lowest significant SAUDPC values, compared with the other rootstocks in the inoculated treatment. Significant intermediate responses were found in the remaining rootstocks, compared with the highest and lowest responses (Figure 1).

### 2.3. Content of Chlorophyll in the Citrus Rootstocks Assayed

The chlorophyll content (SAUCPC) was statistically different among citrus rootstocks and between *R. necatrix* inoculation treatments (*R. necatrix* treatment: F_1624_ = 253.43, and *p* < 0.001; Citrus rootstock: F_11,624_ = 39.19, and *p* < 0.001; *R. necatrix* treatment × Citrus rootstock: F_11,624_ = 6.63, and *p* < 0.001). Carrizo citrange in the control treatment had the greatest chlorophyll content, compared to the other rootstocks in both treatments. By contrast, the inoculated response of CL-5146 displayed the lowest chlorophyll content, with statistical differences, compared with its control and the other rootstocks in both treatments, except with the inoculated treatment of 2247 × 6070-02-2. Only B11R5T60 and B11R5T64 showed similar SAUCPC values with intermediate responses in both treatments; although, no significant differences were detected between inoculated and non-inoculated treatments. Finally, each remaining rootstock displayed higher SAUCPC in the treatment control than in the inoculated treatment, with significant differences between both treatments in each rootstock (Table 1).

### 2.4. Leaf Area Response in Different Citrus Rootstocks

In the case of leaf area, all citrus rootstocks displayed statistical differences among one another and between both treatments (*R. necatrix* treatment: F_1192_ = 134.61, and *p* < 0.001; Citrus rootstock: F_11,192_ = 18.59, and *p* < 0.001; *R. necatrix* treatment × Citrus rootstock: F_11,192_ = 3.94, and *p* < 0.001). First, the greatest leaf area was found in the control treatment of Orange-14, compared with all citrus rootstocks in both treatments, except B11R3T27, B11R3T53 and UFR-1 under control conditions. In the inoculated treatment, Carrizo citrange had the smallest leaf area with significant differences compared with Orange-14, 2247 × 6070-02-2, B11R3T27, B11R3T53 and B11R5T49, and all rootstock responses under control conditions, except B11R5T60 and B11R5T64. Only B11R5T60 and B11R5T64 displayed similar responses in leaf area between them in both treatment conditions, all the other rootstocks each showed statistical differences between inoculated and non-inoculated treatments (Table 2).

### 2.5. Effect of R. necatrix on Biomass Production

#### 2.5.1. Fresh Weight

Statistical differences were detected among the citrus rootstocks tested for the fresh weight of above ground sections (FWAG) (F_1166_ = 6.35, and *p* < 0.001). The highest significant percentage of biomass reduction was observed for rootstocks UFR-5 and CL-5146, compared with the other rootstocks, except Carrizo citrange, UFR-1 and B11R3T53, which, although presenting lower values of biomass reduction, did not differ statistically from the previous treatments. On the other hand, B11R5T60 showed the lowest rate of biomass reduction with statistical differences compared with the rootstocks assayed, except with B11R5T64. The remaining citrus rootstocks (Orange-14, UFR-6, B11R3T27, 2247 × 6070-02-2 and B11R5T49) displayed a reduction percentage ranging between 40.94% and 28.83%, without significant differences among them (Table 3).

In the case of root sections (FWR), significant differences were found among the citrus rootstock (F_1166_ = 17.70, and *p* < 0.001). B11R5T60 displayed the lowest statistical reduction percentage of biomass, together with B11R5T49 and B11R5T64, compared with all the others citrus rootstocks assayed. By contrast, the highest significant reduction rate was found in CL-5146 and B11R3T53, compared with the lowest percentage. In addition, Orange-14 showed an intermediate response with statistical differences, compared with the highest and the lowest percentages. The remaining citrus rootstocks (Carrizo citrange, UFR-5, 2247 × 6070-02-2, B11R3T27, UFR-1 and UFR-6), with ranges between 82% and 75.45%, did not show significant differences among them, compared with the highest response (CL-5146) (Table 3).

#### 2.5.2. Dry Weight

Statistical differences were observed among the citrus rootstocks assayed for the dry weight of above ground sections (DWAG) (F_1166_ = 2.36, and *p* = 0.02). The highest percentage of biomass reduction was observed in UFR-5, CL-5146 and Carrizo citrange, with significant differences only when compared with B11R5T49 and B11R5T60. The latter rootstock showed the lowest response. The remaining rootstocks (B11R3T53, UFR-1, UFR-6, 2247 × 6070-02-2, B11R3T27, Orange-14 and B11R5T64) did not display significant differences on reduction rate among them and the highest response, which ranged from 34.86% to 21.14% (Table 3).

Significant differences were found among the citrus rootstocks for the dry weight of root sections (DWR) (F_1166_ = 11.00, and *p* < 0.001). Carrizo citrange had the highest statistical reduction rate, compared with Orange-14, B11R5T64, B11R5T49, and the lowest response was for B11R5T60. The percentage of reduction for the remaining rootstocks (CL-5146, UFR-5, B11R3T53, UFR-1, UFR-6, 2247 × 6070-02-2 and B11R3T27) ranged between 77.34% and 64.40%, with significant differences, compared with B11R5T64, B11R5T49 and B11R5T60 (Table 3).

## 3. Discussion

In this current study, we successfully evaluated the incidence of white root rot, caused by *R. necatrix*, in different newly-obtained citrus rootstocks. To our knowledge, the effect of this citrus pathogen has not been researched on HLB tolerant citrus rootstocks.

Previous research [5] evaluated the effect of *R. necatrix* artificial inoculations in four citrus rootstocks (Troyer citrange, *P. trifoliata*, *C. macrophylla* and *C. aurantium*), in which the most susceptible one was sour orange (*C. aurantium*) and *P. trifoliata* displayed the highest tolerance response. In our study, none of the assayed new citrus rootstocks contained sour orange in parentage. The new citrus rootstocks, B11R5T64 and B11R5T60, showed the lowest disease incidence and symptoms due to *R. necatrix* artificial inoculations, with similar results to those of the non-inoculated control. Additionally, only these rootstocks revealed similar responses for chlorophyll content and leaf area between the applied treatments (control and inoculated). B11R5T60 had the lowest rate of biomass reduction in all evaluated sections for fresh and dry weight, and B11R5T64 displayed the second lowest reduction in FWAG and FWR. Furthermore, B11R5T64 and B11R5T60 (diploids) possessed the same parentage (Table 4), for which *P. trifoliata* ‘Flying Dragon’ was a direct parent in both rootstocks [66,67]. In the same way, trifoliate orange, as *P. trifoliata*, was reported with resistance against diseases caused by the other soil-borne pathogens, including *Phythophthora* spp. [68]. Other rootstocks, such as Carrizo citrange, possessed direct parentage with *P. trifoliata*, but the resistance to white root rot was not a dominant characteristic for this rootstock in our study. Additionally, B11R5T64 and B11R5T60 manifested the lowest degree of foot rot disease caused by *Phytophthora nicotianae* in a previous work [66].

In addition, Sztejnberg and Madar [5] reported an intermediate response from Troyer citrange (*C. sinensis* ‘Washington’ × *P. trifoliata*) with *R. necatrix* artificial inoculations; however, this citrus rootstock displayed the highest incidence of white root rot by *R. necatrix* natural infection under field conditions. Although we did not evaluate Troyer citrange, this citrus rootstock and Carrizo citrange are often considered identical [69,70]. Additionally, both possess the same parentage from a cross made in the citrus breeding program of USDA at Riverside (California) in 1909 and have the same genus (*X Citroncirus* sp.) in the taxonomy [71,72]. In this present work, Carrizo citrange, next to CL-5146 and UFR-5, were the rootstocks most affected by *R. necatrix* infections in symptoms evaluation, and they displayed wide differences between control and inoculated treatments in symptoms, and in chlorophyll and leaf area measurements for each rootstock. CL-5146 and Carrizo citrange had the lowest chlorophyll content and leaf area, respectively. These three citrus rootstocks all displayed the highest percentages of biomass reduction in all plant sections of fresh and dry weight. However, Carrizo and Troyer citranges were considered tolerant, or having intermediate resistance, to diseases caused by *Phytophthora* spp. In [71,73].

Concerning the seven remaining citrus rootstocks (UFR-6, UFR-1, B11R3T53, 2247 × 6070-02-2, B11R3T27, B11R5T49 and Orange-14), all displayed intermediate responses for disease symptoms and percentage of biomass reduction, and wide differences between both treatments in chlorophyll and leaf area. Orange-14 showed the second lowest symptom rate after B11R5T64 and B11R5T60, and the highest leaf area result of inoculated plants, compared with the other citrus rootstocks. Nevertheless, Orange-14 had the highest incidence of foot rot disease incidence in [66]. Similar symptom responses to white root rot as that shown by Orange-14 were found among B11R5T49 and B11R3T27, which had the highest chlorophyll content in inoculated plants, compared with the other citrus rootstocks, and B11R5T49 was found to be in the second lowest group of biomass reduction.

At the end of the experiment, the isolate Rn452 was isolated from roots of one plant per rootstock belonging to the inoculated treatment group, so as to corroborating that the disease was caused by *R. necatrix*. For the isolation, root pieces were selected because this fungus only infects up to this level and does not invade the vascular system of the plant, to reach the stems or leaves; although the translocation of toxins from *R. necatrix* in the vascular system of plants has been demonstrated [74].

On the other hand, only Orange-14, UFR-1, UFR-5 and UFR-6 were previously studied regarding fruit production and quality with ‘Hamlin’ orange [75]. UFR-5 and Orange-14 had high ‘Hamlin’ orange production in the two trial locations and harvest seasons (2018–2019 and 2019–2020), whereas, UFR-1 and UFR-6 displayed high fruit production in one trial location and intermediate production in the second trial location during both harvest seasons. In addition, these four citrus rootstocks were described as having similarly high fruit production as Carrizo citrange [76], and they, together with B11R3T27, are being evaluated under field conditions for production parameters in two locations in Florida [77]. Up to date, there is no productivity publications for the remaining citrus rootstocks. All these 12 citrus rootstocks were recently established (2021) under field conditions in an experimental plot at “Las Torres” Center of the Andalusian Institute for Agricultural and Fisheries Research and Training (IFAPA), Alcalá del Río, Seville, Spain (37°30′52.52″ N; 5°57′59.66″ W), and grafted with the ‘Lane Late’ cultivar, but without production data. due to tree longevity.

In prior research with other fruit trees, rootstock genetic resistance against *R. necatrix* was found with different levels of success. Thus, Lee et al. [46] screened a total of 177 apple rootstocks, and, after several trials, they obtained five clones with tolerance (2.8%). In persimmon trees, a total of 468 rootstocks were tested, with an achievement percentage of 5.7% for *Diospyros virginiana* and 24.5% for *D. kaki* [51,52]. In the case of avocado, two previous studies were performed; in the first, 13 avocado rootstocks were selected from a total of 4753 (0.3%) [78], and in the second, a success percentage of 1.5% was obtained from 40 selected rootstocks from a total of 2612 [48]. In this last study, the avocado breeding program of IFAPA-Málaga obtained the outstanding candidate “BG83”, which has been used in recent investigations [79]. In our first study of citrus rootstock tolerance to *R. necatrix* infection, we found two promising candidates (B11R5T60 and B11R5T64) out of 12 tested, with a success percentage of 16.7%, higher than apple, *D. virginiana* and avocado, but lower than *D. kaki*. Additionally, B11R5T60 and B11R5T64 were described to have some tolerance to HLB [66]; personal communication, F.G. Gmitter Jr. They could be useful for citrus growers to combat the described diseases under effective and sustainable long-term integrated strategies in producing regions or countries with the presence of, or emergence risk of, their causal agents. These results could be helpful for the research community and breeding programs to improve their plant material in these pathological research fields.

## 4. Materials and Methods

### 4.1. Plant Material and Experimental Design

A total of 216 plants, belonging to twelve different citrus rootstocks, were evaluated in this work, and 11 new citrus rootstocks (6 diploids and 5 tetraploids) from different breeding programs. Carrizo citrange (diploid) was selected as the standard comparative rootstock (Table 4), which is commercially available in Spain under the registered number 16690003 [80]. Twenty-six-month-old plants of this rootstock were provided by Agromillora Group nursery (Subirats, Barcelona, Spain) from previous grown in vitro culture. Each citrus plant was cultivated in a 3 L plastic pot with substrate composed of coconut fiber (80%) and peat (20%), enriched with 5 g of Osmocote ^®^ Pro (16 + 11 + 10 + 2 MgO + trace elements, longevity 12–14 months) per liter of substrate. The experiment was carried out during the spring-summer season of 2022, in a greenhouse (26.00 °C and 64.48% average temperature and relative humidity, respectively) located in the “Las Torres” Center of the Andalusian Institute for Agricultural and Fisheries Research and Training (IFAPA), Alcalá del Río, Seville, Spain (37°30′43.3″ N; 5°57′47.5″ W). After the reception of the plants, they were separated into two treatments [inoculated and control (non-inoculated)], distributed randomly and kept under an acclimation period of two weeks in the greenhouse. For each treatment and rootstock, a total of nine replicates (n = 9) were used. All plants were irrigated thrice per week, depending on water requirements.

**Table 4:** Citrus rootstock evaluated against white root rot disease in this work.

Rootstock	Parentage	Origin	Ref.
Diploids			
Carrizo citrange	*Citrus sinensis* ‘Washington’ × *Poncirus trifoliata*	USDA	[69]
B11R3T27	*P. trifoliata* ‘Flying Dragon’ × *C. paradisi* ‘Duncan’	CREC	[77]
B11R3T53	(*C. reticulata* ‘Cleopatra’ × *C. ichangensis*) × (*C. maxima* × *P. trifoliata*)	CREC	[66]
B11R5T49	*P. trifoliata* ‘Flying Dragon’ × *C. sinensis* ‘Ridge pineapple’	CREC	[66]
B11R5T60	*P. trifoliata* ‘Flying Dragon’ × *C. sinensis* ‘Ridge pineapple’	CREC	[67]
B11R5T64	*P. trifoliata* ‘Flying Dragon’ × *C. sinensis* ‘Ridge pineapple’	CREC	[66]
CL-5146	*C. sunki* × *Citroncirus* spp. ‘Swingle’	CL	[66]
Tetraploids			
Orange-14 *	*C. reticulata* ‘Nova’ + *C. maxima* HBP × *C. reticulata* ‘Cleopatra’ + *P. trifoliata*	CREC	[81]
UFR-1 (Orange-3) *	*C. reticulata* ‘Nova’ + *C. maxima* HBP × *C. reticulata* ‘Cleopatra’ + *P. trifoliata*	CREC	[75,82,83]
UFR-5 (White 4) *	*C. reticulata* ‘Nova’ + *C. maxima* HBP × *C. sinensis* ‘Succari’ + *P. trifoliata*	CREC	[75,84]
UFR-6 **	*C. reticulata* ‘Changsha’ + *P. trifoliata* ‘50-7’	CREC	[75,85]
2247 × 6070-02-2 *	*C. reticulata* ‘Nova’ + *C. maxima* HBP × *C. aurantium*+*Poncirus trifoliata* ‘Flying Dragon’	CREC	[67]

Ref.: references; *: tetrazyg; **: somatic hybrid; +: indicates somatic hybridization (allotetraploid); ×: indicates sexual hybridization (diploid or tetraploid); HBP: ‘Hirado Buntan Pink’ pummelo seedling; CREC: Citrus Research and Education Center (Lake Alfred, FL, USA); CL: Citrolima nursery (Casa Branca, State of São Paulo, Brazil); USDA: United States Department of Agriculture (Riverside, CA, USA).

### 4.2. Fungal Isolate

A highly pathogenic *R. necatrix* isolate (Rn452) [personal communication C.J. López-Herrera and J.M. Arjona-López] was selected and supplied from the fungal collection of Plant Protection Department at Institute for Sustainable Agriculture (IAS), Spanish Research Council (Córdoba, Spain). The fungal species was previously confirmed by IAS, following the procedure described by Arjona-López et al. [86]. Briefly, Rn452 was grown over cellophane membrane on potato dextrose agar (15 mL; PDA; Difco Laboratories, Detroit, Michigan, USA) on Petri plates (90 mm in diameter) at 25 °C for 5 days in darkness (chamber conditions, CC). Then, the DNA was extracted from small portions (approximately 17 mg) of fresh fungus mycelium using the i-genomic Plant DNA Extraction Mini Kit (iNtRON Biotechnology, Inc., Seongnam, Korea). PCR amplification and further sequencing of the Internal Transcribe Spacer (ITS) region of the nuclear rDNA was performed with ITS4 and ITS5 primers [87]. The PCR reaction was mixed in a total volume of 25 µL containing 1.75 ng of DNA, 0.8 mM of dNTPs, 2.5 mM of MgCl_2_, 1X of PCR Buffer, 0.75 µM of each primer and 0.05 U/µL of Horse-Power-Taq DNA polymerase (Canvax Biotech, S.L., Córdoba, Spain). This reaction was amplified in a BT1 Thermocycler (Whatman Biometra, Göttingen, Germany) with an initial step at 95 °C for 3 min, followed by 30 cycles of 30 s at 56 °C, 2 min at 72 °C and 30 s at 95 °C, including a final step for 10 min at 72 °C. The amplified fragments were visualized by electrophoresis in 2% agarose gel stained with RedSafe (iNtRON Biotechnology, Inc., Seongnam, Korea). The PCR product (600 bp) was sequenced by the Molecular Biology on Proteomic Department of Central Research Support Service (SCAI, University of Córdoba, Spain). The raw sequences were edited by Chromas 2.6.4 program (Technelysium Pty Ltd., South Brisbane, Australia), assembled by the DNAMAN 6.0.3.93 program (Lynnon Corporation, San Ramon, CA, USA), and compared with sequences from GenBank, using the BLAST tool (version 2.0, National Center for Biotechnology Information).

### 4.3. Plant Inoculation

The inoculation process was carried out at the beginning of the experiment and followed the method described by Sztejnberg and Madar [5], with slight modifications. Thus, Rn452 was refreshed on 15 mL PDA Petri plates from the supplied fungal culture and incubated at CC. Then, double-sterilized wheat grains were incubated in 1000 mL of Teqler flasks with fungal mycelial disks from the refreshed cultures for three weeks under CC, until a total fungal colonization of the grains was achieved. Finally, the selected plants (inoculated treatment) were inoculated, placing in each root ball (in contact with roots) a portion of 3.75 g of colonized wheat grains per liter of substrate.

### 4.4. Plant Symptoms Evaluation

**Figure 2:**
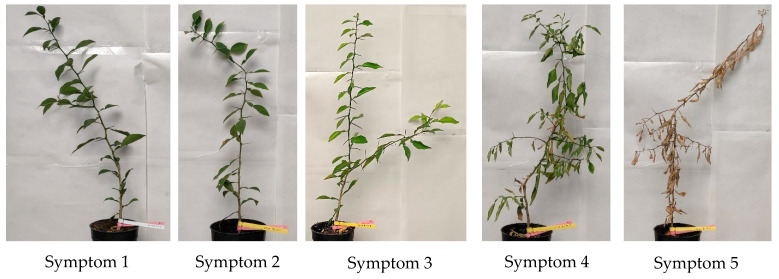
Disease index with a symptom scale of 1–5: 1, healthy plant; 2, first signs of leaf decline and chlorosis on plant; 3, plant with chlorotic and curly leaves; 4, wilted plant with first symptoms of leaf desiccation; and 5, dead plant.

The above-ground symptoms of the disease were recorded for each plant from all treatments considered, using the disease index with a symptoms scale of 1–5, where: 1, healthy plant; 2, first signs of leaf decline and chlorosis on plant; 3, plant with chlorotic and curly leaves; 4, wilted plant with first symptoms of leaf desiccation; and 5, dead plant (Figure 2). These assessments were carried out on two days (every Monday and Thursday) per week, starting from the time of pathogen inoculation until all plants of one inoculated treatment and rootstock died, after a total of 89 days. All the values obtained were used to calculate the standardized area under the disease progress curve (SAUDPC) [88], which increased in the same proportion as the symptom scale.

### 4.5. Evaluation of Leaf Chlorophyll Content

The leaf chlorophyll content was measured for all the nine plant replicates from all treatments using a chlorophyll meter MC-100 (µmol of chlorophyll per m^2^ of leaf; Apogee Instruments Inc., North Logan, UT, USA). The measuring process was performed on three leaves (sub-samples) per plant and repeated once a week (every Wednesday), starting from the time of pathogen inoculation until all plants of one inoculated treatment and rootstock died. The values recorded were used to calculate the standardized area under the chlorophyll progress curve (SAUCPC) [88].

### 4.6. Leaf Area Assessment

At the end of the experiment, all leaves were hand-collected from all the nine plant replicates in both treatments and placed in a labeled paper envelope. The evaluation of all leaves was performed with an area meter LI-3100C (cm^2^; LI-COR Biosciences, Lincoln, NE, USA) per each replicate, separately. After this measurement, each group of leaves were again packaged in paper envelopes.

### 4.7. Biomass

At the end of the experiment, six plant replicates per citrus rootstock and treatment were selected to harvest in three different sections (roots, stem and leaves). Each root section was washed under tap water to remove the substrate, dried over filter paper and kept in a paper envelope. Stem sections were separately collected and kept in a paper envelope. Each group of sections were weighed using a digital scale CB-3000C (g; COBOs precision, L’Hospitalet de Llobregat, Barcelona, Spain). In the case of leaf samples, the same plant replicates selected for root and stem were immediately weighed after the leaf area assessment. After recording fresh weight (FW), each one was placed in the same paper envelope, dried in an oven at 60 °C for 48 h and weighed again to obtain the dry weight (DW). Then, the percentage of biomass reduction (PBR; %) was calculated with the data for FW and DW in each plant section per each sample, according to Vincent’s equation [89]:

$$\mathrm{PBR}\;\left(\%\right)=\frac{\mathrm{CW}-\mathrm{TW}}{\mathrm{CW}}\times 100$$

where: CW is weight (g) of control treatment plants (non-inoculated) averaged across six replicates from fresh weight per plant section; TW is weight (g) of inoculated plants from fresh weight per plant section.

### 4.8. Fungal Isolation from Inoculated Plants

At the end of the experiment, a traditional method of fungal isolation was performed to confirm the presence of *R. necatrix* in inoculated plants [32]. One of the three remaining plants for each rootstock in the inoculated treatment was selected. Next, the plant roots were separately collected, washed under tap water and cut into small pieces which were placed in glass flasks. Under laboratory conditions, the root pieces were surface-sterilized with sodium hypochlorite (8 g L^−1^ of active chlorine) for 3 min, thrice washed with sterile distilled water for 3 min, dried on sterile filter paper and transferred to Petri dishes containing 15 mL of acidified PDA with lactic acid (0.2%). The cultures were incubated under CC for four days until the mycelia grew; then, they were transferred to 15 mL PDA Petri plates to check the morphological structures.

### 4.9. Statistical Analysis

Values obtained from SAUDPC, FW and DW [above ground (leaves + stems) and roots sections] were analyzed as one-way ANOVA (Analysis of variance); whereas, values of SAUCPC and leaf area were analyzed as two-way ANOVA using the free software R version 4.1.2 [90]. Means separation were obtained using LSD–Fisher test (*p* < 0.05) [91] through the “agricolae” package [92]. Figure 1 was also plotted with the same free software version using the “ggplot2” package [93].

## Figures and Tables

**Table 1:** Mean standardized area under the chlorophyll progress curve (SAUCPC) ± standard error (SE) due to the effect of *R. necatrix* (inoculated and non-inoculated) on leaves in 12 citrus rootstocks (Carrizo citrange, B11R3T27, B11R3T53, B11R5T49, B11R5T60, B11R5T64, CL-5146, Orange-14, UFR-1, UFR-5, UFR-6 and 2247 × 6070-02-2).

	Effect of *R. necatrix*	
**Rootstock**	**Inoculated**	**Control (Non-Inoculated)**
Carrizo citrange	540.36 ± 29.44 ^defg^	816.03 ± 14.34 ^a^
B11R3T27	558.89 ± 24.89 ^def^	733.27 ± 17.68 ^b^
B11R3T53	488.93 ± 23.00 ^ghi^	558.39 ± 19.64 ^def^
B11R5T49	563.07 ± 25.43 ^de^	652.77 ± 17.09 ^c^
B11R5T60	432.57 ± 35.77 ^ij^	443.48 ± 32.64 ^hij^
B11R5T64	415.45 ± 31.65 ^j^	438.08 ± 30.48 ^hij^
CL-5146	253.37 ± 15.02 ^l^	472.50 ± 19.64 ^ghij^
Orange-14	413.12 ± 23.05 ^j^	577.84 ± 15.70 ^d^
UFR-1	503.04 ± 21.78 ^efgh^	724.23 ± 16.21 ^b^
UFR-5	339.68 ± 20.73 ^k^	562.60 ± 21.61 ^de^
UFR-6	491.03 ± 34.29 ^fghi^	736.13 ± 27.37 ^b^
2247 × 6070-02-2	290.85 ± 18.99 ^kl^	484.22 ± 30.66 ^ghi^

Values with different letters denote statistical differences among citrus rootstocks and between treatments assayed by LSD-Fisher’s test (*p* < 0.05).

**Table 2:** Mean leaf area response (cm^2^) ± standard error (SE), due to the effect of *R. necatrix* (inoculated and non-inoculated), in 12 citrus rootstocks (Carrizo citrange, B11R3T27, B11R3T53, B11R5T49, B11R5T60, B11R5T64, CL-5146, Orange-14, UFR-1, UFR-5, UFR-6 and 2247 × 6070-02-2).

	Effect of *R. necatrix*	
**Rootstock**	**Inoculated**	**Control (Non-Inoculated)**
Carrizo citrange	64.29 ± 13.78 ^h^	355.37 ± 15.24 ^efg^
B11R3T27	538.29 ± 108.22 ^cde^	1264.55 ± 200.95 ^ab^
B11R3T53	525.99 ± 87.03 ^cde^	1108.15 ± 115.55 ^ab^
B11R5T49	457.17 ± 92.95 ^def^	777.96 ± 104.30 ^c^
B11R5T60	236.20 ± 40.98 ^fgh^	192.99 ± 25.43 ^gh^
B11R5T64	174.74 ± 29.94 ^gh^	181.17 ± 15.13 ^gh^
CL-5146	110.77 ± 17.06 ^gh^	695.82 ± 117.99 ^cd^
Orange-14	684.86 ± 130.25 ^cd^	1337.15 ± 173.95 ^a^
UFR-1	292.19 ± 79.69 ^efgh^	1082.67 ± 66.34 ^ab^
UFR-5	124.19 ± 28.18 ^gh^	676.36 ± 65.48 ^cd^
UFR-6	297.11 ± 81.02 ^efgh^	674.18 ± 105.60 ^cd^
2247 × 6070-02-2	546.82 ± 121.05 ^cde^	1070.27 ± 87.79 ^b^

Values with different letters denote statistical differences among citrus rootstocks and between treatments assayed by LSD–Fisher’s test (*p* < 0.05).

**Table 3:** Mean Percentage of biomass reduction (%) ± standard error (SE) of fresh (FW) and dry weight (DW) on above ground sections (AG; leaves + stems) and root sections (R), due to the effect of *R. necatrix*, in 12 citrus rootstocks (Carrizo citrange, B11R3T27, B11R3T53, B11R5T49, B11R5T60, B11R5T64, CL-5146, Orange-14, UFR-1, UFR-5, UFR-6 and 2247 × 6070-02-2).

Rootstock	FWAG ± SE	FWR ± SE	DWAG ± SE	DWR ± SE
Carrizo citrange	61.74 ± 3.38 ^ab^	82.00 ± 1.36 ^ab^	40.00 ± 5.72 ^a^	78.46 ± 2.03 ^a^
B11R3T27	33.73 ± 8.56 ^de^	77.75 ± 1.86 ^ab^	24.08 ± 9.66 ^ab^	64.40 ± 3.33 ^ab^
B11R3T53	48.44 ± 5.02 ^abcd^	83.63 ± 1.84 ^a^	34.86 ± 4.74 ^ab^	75.00 ± 3.60 ^ab^
B11R5T49	28.83 ± 9.91 ^de^	52.05 ± 5.81 ^c^	18.19 ± 7.44 ^bc^	45.23 ± 6.14 ^c^
B11R5T60	5.80 ± 3.48 ^f^	22.26 ± 9.27 ^d^	2.77 ± 2.46 ^c^	23.39 ± 9.85 ^d^
B11R5T64	22.53 ± 8.02 ^ef^	42.33 ± 6.84 ^c^	21.14 ± 7.90 ^abc^	46.80 ± 6.18 ^c^
CL-5146	67.08 ± 3.65 ^a^	83.97 ± 1.64 ^a^	41.55 ± 6.54 ^a^	77.34 ± 1.73 ^ab^
Orange-14	40.94 ± 10.86 ^bcde^	70.36 ± 4.12 ^b^	23.52 ± 10.78 ^abc^	63.90 ± 4.40 ^b^
UFR-1	56.13 ± 4.71 ^abc^	75.64 ± 2.40 ^ab^	32.85 ± 5.13 ^ab^	69.22 ± 2.51 ^ab^
UFR-5	67.53 ± 4.31 ^a^	81.21 ± 2.90 ^ab^	41.79 ± 5.14 ^a^	76.37 ± 4.51 ^ab^
UFR-6	36.97 ± 9.94 ^cde^	75.45 ± 2.87 ^ab^	25.92 ± 9.28 ^ab^	67.92 ± 3.23 ^ab^
2247 × 6070-02-2	33.33 ± 11.08 ^de^	78.95 ± 6.47 ^ab^	25.15 ± 9.12 ^ab^	68.88 ± 6.17 ^ab^

Values in columns with different letters denote statistical differences among the citrus rootstocks assayed by LSD-Fisher’s test (*p* < 0.05).

## Data Availability

Not applicable.
